# Clinical-Pathological Characteristics and Prognosis of a Cohort of Oesophageal Cancer Patients: a Competing Risks Survival Analysis

**DOI:** 10.2188/jea.JE20140118

**Published:** 2015-03-05

**Authors:** Elena Rodríguez-Camacho, Salvador Pita-Fernández, Sonia Pértega-Díaz, Beatriz López-Calviño, Teresa Seoane-Pillado

**Affiliations:** Clinical Epidemiology and Biostatistics Research Group, Instituto de Investigación Biomédica de A Coruña (INIBIC), Complexo Hospitalario Universitario de A Coruña (CHUAC), SERGAS, Universidade da Coruña, Coruña, Spain

**Keywords:** oesophageal neoplasms, therapeutics, survival, follow-up studies

## Abstract

**Background:**

To determine the clinical course, follow-up strategies, and survival of oesophageal cancer patients using a competing risks survival analysis.

**Methods:**

We conducted a retrospective and prospective follow-up study. The study included 180 patients with a pathological diagnosis of oesophageal cancer in A Coruña, Spain, between 2003 and 2008. The Kaplan-Meier methodology and competing risks survival analysis were used to calculate the specific survival rate. The study was approved by the Ethics Review Board (code 2011/372, CEIC Galicia).

**Results:**

The specific survival rate at the first, third, and fifth years was 40.2%, 18.1%, and 12.4%, respectively. Using the Kaplan-Meier methodology, the survival rate was slightly higher after the third year of follow-up. In the multivariate analysis, poor prognosis factors were female sex (hazard ratio [HR] 1.94; 95% confidence interval [CI], 1.24–3.03), Charlson’s comorbidity index (HR 1.17; 95% CI, 1.02–1.33), and stage IV tumours (HR 1.70; 95% CI, 1.11–2.59). The probability of dying decreased with surgical and oncological treatment (chemotherapy and/or radiotherapy) (HR 0.23; 95% CI, 0.12–0.45). The number of hospital consultations per year during the follow-up period, from diagnosis to the appearance of a new event (local recurrences, newly appeared metastasis, and newly appeared neoplasias) did not affect the probability of survival (HR 1.03; 95% CI, 0.92–1.15).

**Conclusions:**

The Kaplan-Meier methodology overestimates the survival rate in comparison to competing risks analysis. The variables associated with a poor prognosis are female sex, Charlson’s comorbidity score and extensive tumour invasion. Type of follow-up strategy employed after diagnosis does not affect the prognosis of the disease.

## INTRODUCTION

Oesophageal cancer is the eighth-most common cancer worldwide.^1^ In the 27-member European Union, a total of 33 013 new cases were estimated in 2008 (age-standardised rate for world population [ASR{W}]: 3.5 per 100 000).^1^ In relation to the rest of Europe, Spain is at a midway point in terms of occurrence (ASR[W]: 2.8 per 100 000). Within Spain, this cancer occurs at a higher rate in the country’s northern regions than in southern ones.^2^

At the worldwide level, oesophageal cancer is the sixth-most frequent cause of death by cancer. In 2008, the global age-standardised rate of cancer-related mortality was 2.9 per 100 000 inhabitants in the European Union and 2.3 per 100 000 in Spain. The mortality rate, like the occurrence rate, is higher in Spain’s northern regions.^3^

According to the European Cancer Registry EUROCARE-4 study,^4^ the average European survival rate is close to 35% at one year and close to 10% at 5 years, and survival is strongly associated with the clinical stage of the tumour and the treatment the patient receives.^5^^–^^7^ There is little consensus when it comes to defining the follow-up protocols for these patients.^8^ Furthermore, to the best of our knowledge, there are no observational or experimental studies that have investigated the role of the different follow-up strategies on these patients’ prognosis.

With regard to analysing the survival rate, competing risk models are the most suitable for analysing the behaviour of subjects who may die for different reasons. However, when applied in the presence of competitive risks, the usual techniques for analysing the time until the event, such as the Kaplan-Meier methodology, produce biased results.^9^^,^^10^ By using specific techniques, it is possible to reduce bias so that results can be correctly interpreted.

Therefore, the geographic variability in the occurrence and mortality rates, the existence of different risk factors associated with the low survival rate for these kinds of tumours, and the lack of consensus with regard to optimum follow-up strategies justify the undertaking of this study. The aim of the study was to identify the epidemiology of oesophageal cancer in the area of A Coruña, Spain, the supportive process applied to these patients, and their prognosis, using the competing risks methodology.

## METHODS

A total of 234 patients were included in the study, with anatomical-pathological confirmation of cancer of the oesophagus diagnosed at the University Hospital Complex in A Coruña, Spain between the 1st of January, 2003, and the 31st of December, 2008. A retrospective review of the patients’ clinical records was carried out together with a prospective follow-up until the 31st of January, 2012, in order to guarantee a minimum follow-up period of 3 years. The study excluded prevalent or recurring cases, subjects with multiple or metastatic cancers, or those that had been treated and/or diagnosed at other hospitals. After exclusions, the final study sample comprised 180 patients.

### Measurements

We collected information on the socio-demographic variables of the patient, their personal backgrounds, comorbidity variables using Charlson’s comorbidity index, the symptoms present at diagnosis, location of the tumour, histopathologic cell type, and tumour stage (TNM, seventh edition).^11^^,^^12^ As data on risk factors and symptoms were collected retrospectively from electronic hospital clinical records, we registered data that were indicated in the clinical records but could not quantify the frequency and amount of cigarette or alcohol consumption, nor the number of kilograms lost during the previous months before the diagnosis.

We also collected data on the treatment received (surgery, chemotherapy, and radiotherapy), as well as the visits and tests carried out during the follow-up period: consultations and hospital stays, endoscopies, computer-aided tomography scans, and thorax X-rays. Also, the presence of follow-up events, including newly appeared local recurrences, metastasis, and neoplasias, was studied. For all dead patients, cause of death was obtained from the Galician Mortality Registry (General Directory of Public Health, Xunta de Galicia, Spain), according to the 21 diagnostic categories of the 10th revision of the International Classification of Diseases.

### Sample size justification

The study included a total of 180 patients. This sample size makes it possible to detect as significant a hazard ratio of 1.6 or more, with a prevalence of exposure of 50% and a censored data percentage of 20% (security: 95%; statistical power: 80%).

### Statistical analysis

A descriptive study was made of the variables that were obtained. The specific survival rate was calculated using the Kaplan-Meier methodology and competing risks survival analysis. The accumulated occurrence of dying as a result of oesophageal cancer during the follow-up period was estimated, considering death as a result of other causes as a competitive event, using the method proposed by Kalbfleisch and Prentice.^13^ The accumulated occurrence of death due to oesophageal cancer according to different characteristics was compared using the test proposed by Gray.^14^ Finally, in order to identify which characteristics were associated with the risk of dying as a result of oesophageal cancer, a multivariate analysis was carried out using the model proposed by Fine and Gray.^15^ All of the tests were carried out bilaterally, considering values of *P* < 0.05 as significant. The analyses were carried out using the programmes Epidat 3.1 (Xunta de Galicia, Santiago de Compostela, Spain), SPSS 19.0 (IBM Company, Chicago, IL, USA), and R 2.15.1 (Free Software Foundation, Boston, MA, USA).

### Ethics

The study was carried out according to the principles laid down in the Declaration of Helsinki and ensuring compliance with Spanish Decree 29/2009, which regulates the use of and access to electronic medical records. Confidentiality was maintained in accordance with the current Spanish Data Protection Law (15/1999). The study received written approval from the regional Ethics Committee for Clinical Research (code 2011/372 CEIC Galicia).

## RESULTS

### Characteristics of the patients studied

Table 1 shows the baseline characteristics, comorbidities, and cause of death of the patients. The median age was 64.5 years, 87.8% of the sample subjects were male, 58.6% had a body mass index (BMI) within normal range, and 11.4% were obese. The most frequent symptom reported was dysphagia (82.0%), followed by weight loss (49.4%). Regarding tumour stage, 46.8% of the tumours were moderately differentiated, 38.0% were poorly differentiated, and 28.9% had metastasis at the time of diagnosis (Table 2).

**Table 1.** Baseline characteristics, comorbidity, and cause of death of the patients studied

	***n***	**Mean**	**SD**	**Median**	**95% CI (Mean)**
**Age (years)**	180	64.2	11.2	64.5	62.6–65.9
**Gender**	***n***	**%**			**95% CI**
Male	158	87.8			82.7–92.8
Female	22	12.2			7.2–17.3

	***n***	**Mean**	**SD**	**Median**	**95% CI (Mean)**

**BMI (kg/m^2^)**	70	24.4	4.0	24.2	23.5–25.4

**Personal background**	***n***	**%**			**95% CI**

Smoking	139	77.2			70.8–83.6
Alcohol consumption	101	56.1			48.6–63.6
Smoking and alcohol consumption	89	49.4			41.9–57.0
Gastro-oesophageal reflux	40	22.2			15.9–28.6

	***n***	**Mean**	**SD**	**Median**	**95% CI (Mean)**

**Charlson’s comorbidity index**	180	1.2	1.3	1.0	1.0–1.4
**Charlson’s comorbidity index age adjusted**	180	3.2	1.8	3.0	2.9–3.5

**Charlson’s comorbidity pathologies**	***n***	**%**			**95%CI**

Myocardial Infarction	5	2.8			0.9–6.4
Congestive Heart Failure	4	2.2			0.6–5.6
Peripheral Vascular Disease	11	6.1			2.3–9.9
Cerebrovascular Disease	5	2.8			0.9–6.4
Dementia	1	0.6			0.0–3.1
Chronic Obstructive Pulmonary Disease	53	29.4			22.5–36.4
Connective Tissue Disease	0	0.0			0.0–2.0
Peptic Ulcer Disease	10	5.6			1.9–9.2
Liver Disease	13	7.2			3.2–11.3
Diabetes Mellitus uncomplicated	29	16.1			10.5–21.8
Hemiplegia	0	0.0			0.0–2.0
Moderate to Severe Chronic Kidney Disease	4	2.2			0.6–5.6
Diabetes Mellitus end-organ damage	1	0.6			0.0–3.1
Solid Tumor	21	11.7			6.7–16.6
Leukemia	2	1.1			0.1–4.0
Malignant Lymphoma	1	0.6			0.0–3.1
Liver Disease (moderate to severe)	9	5.0			1.5–8.5
Metastatic Solid Tumor	0	0.0			0.0–2.0
AIDS	0	0.0			0.0–2.0

**Cause of death**	***n***	**%**			**95% CI**

Oesophageal cancer-related mortality	150	94.9			91.2–98.7
Non oesophageal cancer-related mortality	8	5.1			1.3–8.8
Myocardial Infarction	2	25.0			3.2–65.1
Pulmonary embolism	1	12.5			0.3–52.7
Pulmonary edema	1	12.5			0.3–52.7
Septicemia	1	12.5			0.3–52.7
Chronic Obstructive Pulmonary Disease	1	12.5			0.3–52.7
Malignant neoplasm of other sites	1	12.5			0.3–52.7
Multiple independent malignancy	1	12.5			0.3–52.7

BMI, body mass index; CI, confidence interval; SD, standard deviation.

**Table 2.** Tumour characteristics, TNM classification, and treatment options of the patients studied

	*n*	%	95% CI
Tumour location			
Cervical	15	8.3	4.0–12.7
Upper thoracic	37	20.6	14.4–26.7
Middle thoracic	58	32.2	25.1–39.3
Lower thoracic	54	30.0	23.0–37.0
Distal oesophagus	16	8.9	4.5–13.3
Histopathologic cell type			
Squamous-cell carcinoma	147	81.7	75.7–87.6
Adenocarcinoma	32	17.8	11.9–23.6
Malignant tumor of unknown histology	1	0.6	0.0–3.1
TNM classification			
Stages 0-III	128	71.1	64.2–78.0
Stage IV	52	28.9	22.0–35.8
Treatment			
No treatment	38	21.1	14.9–27.4
Surgery	42	23.3	16.9–29.8
Chemotherapy and/or Radiotherapy	65	36.1	28.8–43.4
Surgery and Chemotherapy and/or Radiotherapy	35	19.4	13.4–25.5

CI, confidence interval.

### Treatment

Treatments involved chemotherapy and/or radiotherapy exclusively in 36.1%, surgery as the sole treatment in 23.3%, and a combination of both in 19.4% (Table 2). In the case of patients who only received surgery, resection was carried out for curative purposes in 74%. Both chemotherapy and radiotherapy were mainly applied for palliative purposes.

### Prognosis

The specific survival rate at 1, 3, and 5 years after diagnosis obtained with the Kaplan-Meier methodology was 39.9%, 19%, and 15%, respectively, while respective survival rates according to competing risks survival analysis were 40.2%, 18.1%, and 12.4% (Table 3).

**Table 3.** Univariate analysis of variables associated or not with cancer-related mortality during the follow-up and survival rate with Kaplan-Meier and competing risks analysis methods

	Oesophageal cancer-related mortality										

	No				Yes				*P*	HR	95% CI
	
	*n*	Mean	SD	Median	*n*	Mean	SD	Median			
Age (years)	28	63.7	8.8	65.0	150	64.3	11.6	64.0	0.170	1.01	(0.99–1.02)
Charlson’s comorbidity index age adjusted	28	2.7	1.3	3.0	150	3.3	1.9	3.0	0.002	1.14	(1.05–1.23)

	Probability of mortality (%) (Oesophageal cancer-related)								*P*	HR	95% CI

	6 months		1 year		3 years		5 years	

Gender											
Male	33.1		56.9		78.2		81.7		—	1	—
Female	47.6		76.2		90.5		95.2		0.050	1.63	(1.00–2.64)
Weight loss											
No	24.8		46.4		72.7		80.5		—	1	—
Yes	44.8		72.4		87.4		87.4		0.002	1.68	(1.20–2.34)
Histopathologic cell type											
Squamous-cell carcinoma	31.0		55.2		77.2		80.9		—	1	—
Adenocarcinoma	53.3		76.7		90.0		93.3		0.021	1.68	(1.08–2.62)
TNM classification											
Stages 0-III	27.0		49.5		73.5		78.1		—	1	—
Stage IV	53.9		82.7		94.2		96.2		<0.001	2.38	(1.63–3.46)
Treatment											
No treatment	73.0		97.3		—		—		—	1	—
Surgery	31.7		59.6		69.7		73.9		<0.001	0.22	(0.12–0.38)
Chemotherapy and/or Radiotherapy	27.7		53.8		83.1		84.6		<0.001	0.26	(0.17–0.39)
Surgery and Chemotherapy and/or Radiotherapy	11.4		28.6		62.9		74.8		<0.001	0.17	(0.10–0.27)

	Survival Rate (Oesophageal cancer-related)										

			1 year		3 years		5 years				

Kaplan-Meier survival rate			39.9%		19.0%		15.0%				
Competing risks survival rate			40.2%		18.1%		12.4%				

CI, confidence interval; HR, hazard ratio; SD, standard deviation.

At 1 year from diagnosis, the probability of dying as a result of the cancer was 59.2%, with the probability of dying from other causes being 0.6% (Figure). At 5 years from diagnosis, the probability of dying as a result of the cancer rose to 83.4% and the probability of dying from other causes was 4.2%; therefore, the probability of survival was reduced to 12.4%.

**Figure.**
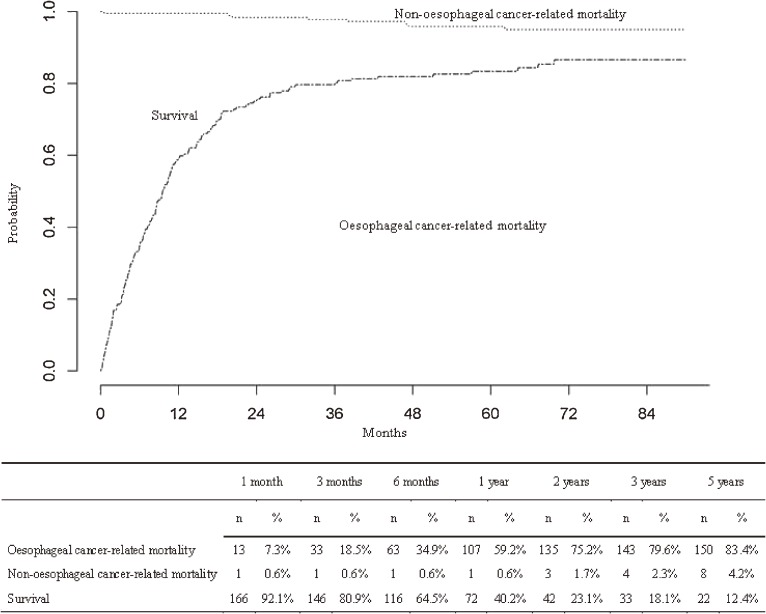
Prognosis of patients with oesophageal cancer after diagnosis

The variables in the univariate analysis that were significantly associated with the probability of dying during the follow-up period were: gender, Charlson’s comorbidity index, presence of weight loss, histopathological cell type, tumour stage, and type of treatment (Table 3). The specific probability of dying was increased among females (hazard ratio [HR] 1.63; 95% CI, 1.00–2.64), those with a higher score on age-adjusted Charlson’s comorbidity index (HR 1.14; 95% CI, 1.05–1.23), and among those with weight loss on diagnosis (HR 1.68; 95% CI, 1.20–2.34). The histopathologic cell type with the highest mortality rate was adenocarcinoma (HR 1.68; 95% CI, 1.08–2.62). In turn, those with stage IV tumours had a higher mortality rate than those in earlier stages (0-III) (HR 2.38; 95% CI, 1.63–3.46).

The patients who had received some kind of treatment had a lower probability of dying. Those who had received a combination of surgical and oncological treatment (chemotherapy and/or radiotherapy) had the lowest probability of dying (HR 0.17; 95% CI, 0.10–0.27), followed by those who had only received surgical treatment (HR 0.22; 95% CI, 0.12–0.38).

No association was found between the probability of dying during the follow-up period and the following variables: year of diagnosis, age, BMI, personal background (smoking, regular alcohol consumption, gastro-oesophageal reflux, achalasia, and a family history of cancer), presence of dysphagia on diagnosis, or tumour location.

According to the final adjustment by multivariate competing risks analysis, we found that the variables with an independent effect to predict mortality are gender, Charlson’s comorbidity index, and tumour stage (Table 4). In order to adjust for age as a clinically relevant and confounding variable and avoid over-adjustment, we included in the model the crude Charlson’s comorbidity index. The specific probability of dying from oesophageal cancer was increased among females (HR 1.94; 95% CI, 1.24–3.03), those with a higher score on Charlson’s comorbidity index (HR 1.17; 95% CI, 1.02–1.33), and in those with stage IV tumours at the time of diagnosis (HR 1.70; 95% CI, 1.11–2.59). Having received some type of treatment improved the prognosis, with a greater impact in cases that received a combination of surgical and oncological treatment (HR 0.23; 95% CI, 0.12–0.45). Furthermore, no significant effect was found when the interaction between TNM classification and type of treatment was added to the multivariate model (*P* = 0.600).

**Table 4.** Multivariate analysis of oesophageal cancer-related mortality adjusting for different variables

Variables	B	SE	*P*	HR	95% CI
Gender (Female vs. Male)	0.660	0.229	**0.004**	1.94	(1.24–3.03)
Age (years)	−0.006	0.007	0.380	0.99	(0.98–1.01)
Charlson’s comorbidity index	0.153	0.066	**0.021**	1.17	(1.02–1.33)
Weight loss	0.301	0.185	0.100	1.35	(0.94–1.94)
Histopathologic cell type (AC vs. SCC)	0.434	0.233	0.063	1.54	(0.98–2.44)
TNM classification (IV vs. 0-III)	0.529	0.216	**0.014**	1.70	(1.11–2.59)
Treatment					
Surgery	−1.305	0.314	**<0.001**	0.27	(0.15–0.50)
Chemotherapy and/or Radiotherapy	−1.076	0.249	**<0.001**	0.34	(0.21–0.56)
Surgery and Chemotherapy and/or Radiotherapy	−1.466	0.340	**<0.001**	0.23	(0.12–0.45)

AC, adenocarcinoma; B, regression coefficient; CI, confidence interval; HR, hazard ratio; SCC, squamous cell carcinoma; SE, standard error.

### Follow-up

The total length of follow-up was 3221.8 months (268.5 years), with a mean of 9.4 months per patient. The most frequently detected events during follow-up were newly appeared metastasis (19.0%)—mainly located in the lungs (48.5%)—and local tumour recurrences (16.7%) (Table 5).

**Table 5.** Appearance of new events during the follow-up

	*n*	%
Local tumor recurrence	29	16.7
Newly appeared metastases	33	19.0
Newly appeared neoplasias	5	2.9

In those patients who underwent curative surgery, the probability of being diagnosed with a tumour recurrence (local tumour recurrence, distant recurrence, or both) during the first year after the surgery was 21.1%, while 25.5% of the patients died without presenting any recurrence. Therefore, 53.4% of the patients who underwent curative surgery were alive and remained disease-free 1 year after the surgery. Similarly, the probability of tumour recurrence 5 years after the surgery was 52.4%, while 29.6% of the patients died without presenting any recurrence. Therefore, only 18% of the patients were alive and disease-free 5 years after curative surgery.

Considering that the follow-up strategy is more intensive once an event occurs during the follow-up period, we studied the follow-up procedure carried out with patients who underwent curative surgery from the time of diagnosis until a new event occurred (local tumour recurrence, newly appeared metastasis, or newly appeared neoplasias) (Table 6). The mean number of hospital consultations per year of follow-up for these patients was 4.1 (standard deviation, 4.2) consultations/year, with a median of 3.1 consultations per year of follow-up (Table 6).

**Table 6.** Follow-up strategies, from diagnosis to the appearance of a new event, in patients who underwent curative intent surgery

	Mean	SD	Median	IQR
Number of hospital consultations/patient/year	4.1	4.2	3.1	0.7–5.9
Number of endoscopies/patient/year	1.3	2.4	0.3	0.0–1.4
Number of thorax X-rays/patient/year	8.8	26.0	2.3	0.3–8.1
Number of CAT scans/patient/year	1.4	4.9	0.0	0.0–0.9
Number of hospital stays/patient/year	1.9	2.4	1.0	0.0–3.0

CAT, computer-aided tomography; IQR, inter-quartile range; SD, standard deviation.

When we take into account gender, age, Charlson’s comorbidity index, weight loss, histopathological cell type, TNM stage, treatment carried out, and number of consultations per follow-up year, in the interval between the diagnosis until an event occurs, we see that the consultations carried out during this period do not substantially alter the likelihood of survival (*P* = 0.640; HR 1.03; 95% CI, 0.92–1.15).

## DISCUSSION

In Spain, the occurrence of oesophageal cancer is at a midway point with regard to the rest of Europe.^1^ It is more frequent in men^16^^–^^18^ and usually appears between the ages of 55 and 70 years,^6^^,^^19^^–^^21^ and the most common symptom associated with its appearance is dysphagia.^22^ The results of the present study confirm these findings and are in line with those of other previously published series.^19^^,^^20^^,^^23^^,^^24^

With regard to the specific survival rate for these types of tumours, data published from the EUROCARE-4^4^ study, which included 48 353 cases from 23 European countries, reveal a mean estimated survival rate of 35%, 14.1%, and 9.6% at the first, third, and fifth years of follow-up, respectively, highlighting the low survival rate associated with these tumours. These data also concur with those published in countries such as Canada^25^ or Iran,^20^ which reported 5-year survival rates of 8.8% and 12%, respectively. Series published in Europe, such as those in Sweden,^6^ England,^26^ and France^27^ found similar figures, with 5-year survival rates between 9.3%–13.1%, 3.2%–9.8%, and 9%–14%, respectively. In our study, the 5-year survival rate was 12.4%. It is important to note that we used the competing risks survival analysis in our study, which is suitable for analysing the behaviour of a person who may die as a result of different causes. Using the Kaplan-Meier methodology, the results obtained for our sample slightly overestimated the survival rate from the third year onwards. The patients in this cohort mainly die as a result of the illness in question, and so for this reason the differences found in the survival rate between the Kaplan-Meier methodology and competing risks survival analysis are very low. Despite this, the competing risks survival analysis method is the most suitable for analysing the specific survival rate in the presence of other causes of death. This overestimation of the survival rate using the Kaplan-Meier methodology in comparison to competing risks survival analysis has previously been described in the literature.^9^^,^^10^

With regard to the factors associated with survival, most studies have indicated that women have higher survival rates than men,^5^^,^^19^^,^^25^^,^^27^ although in some studies these differences were not significant.^20^ In a study of patients from the Donostia Hospital in the Basque Country, Spain,^23^ the mean survival rate in women was lower, as in our study, although the differences were not significant. In our study, we found that the patient’s number and severity of comorbidities, assessed using Charlson’s comorbidity index, is significantly associated with the likelihood of dying as a result of the tumour. In a recent study in the Netherlands,^19^ having comorbidities was associated with poor survival, as in our study, although no significant differences were found.

Amongst the limitations to the study, we did not study any molecular marker that could play an important role in the prognosis of oesophageal cancer and which, in combination with other parameters such as tumour stage, would help to identify and predict the survival rate from these tumours, as well as to individually adapt the treatment to each patient, as recently described in the literature.^28^^,^^29^ Although our sample only consisted of 180 patients, we were able to compare the consistency of our results with larger series published internationally,^5^^–^^7^^,^^20^^,^^23^^,^^24^^,^^30^^–^^32^ in which the tumour stage and treatment received by the patient were the main prognostic factors for survival.

This study reveals how the different follow-up strategies used (visits and tests carried out) in patients where the intention is to apply curative treatment until a new event occurs do not alter their survival rate. This finding is in agreement with the lack of consensus at the international level^8^^,^^33^^,^^34^ in terms of defining the follow-up protocols for these types of tumours. Furthermore, we did not find any studies in the literature that describe the follow-up process applied to these patients and its possible effect on their survival rate. In The European Society for Medical Oncology’s guidelines for the diagnosis, treatment, and monitoring of oesophageal cancer for 2013,^35^ the group concluded that, with the exception of patients who could be candidates for rescue surgery after a failed endoscopic resection or definitive chemo-radiotherapy, there is no evidence that regular follow-up after the initial therapy impacts the final outcome.

In conclusion, the specific survival rate detected in our study was low and coincided with figures published at the international level. Our results confirm that studying the survival rate using the Kaplan-Meier methodology overestimates the survival rate in comparison to competing risks survival analysis. We also found that the different follow-up strategies used after diagnosing illness in patients who are surgically treated with the intention to cure do not alter the prognosis.
